# Toward Systems Models for Obesity Prevention: A Big Role for Big Data

**DOI:** 10.1093/cdn/nzac123

**Published:** 2022-07-30

**Authors:** Adele R Tufford, Christos Diou, Desiree A Lucassen, Ioannis Ioakimidis, Grace O'Malley, Leonidas Alagialoglou, Evangelia Charmandari, Gerardine Doyle, Konstantinos Filis, Penio Kassari, Tahar Kechadi, Vassilis Kilintzis, Esther Kok, Irini Lekka, Nicos Maglaveras, Ioannis Pagkalos, Vasileios Papapanagiotou, Ioannis Sarafis, Arsalan Shahid, Pieter van ’t Veer, Anastasios Delopoulos, Monica Mars

**Affiliations:** Division of Human Nutrition and Health, Wageningen University and Research, Wageningen, Netherlands; Department of Informatics and Telematics, Harokopio University of Athens, Athens, Greece; Division of Human Nutrition and Health, Wageningen University and Research, Wageningen, Netherlands; Department of Biosciences and Nutrition, Karolinska Institute, Stockholm, Sweden; W82GO Child and Adolescent Weight Management Service, Children's Health Ireland at Temple Street, Dublin, Ireland; Division of Population Health Sciences, School of Physiotherapy, Royal College of Surgeons in Ireland University for Medicine and Health Sciences, Dublin, Ireland; Department of Electrical and Computer Engineering, Aristotle University of Thessaloniki, Thessaloniki, Greece; Division of Endocrinology, Metabolism, and Diabetes, First Department of Pediatrics, National and Kapodistrian University of Athens Medical School, “Aghia Sophia” Children's Hospital, Athens, Greece; Division of Endocrinology and Metabolism, Center for Clinical, Experimental Surgery and Translational Research, Biomedical Research Foundation of the Academy of Athens, Athens, Greece; College of Business, University College Dublin, Dublin, Ireland; Geary Institute for Public Policy, University College Dublin, Dublin, Ireland; COSMOTE Mobile Telecommunications, Athens, Greece; Division of Endocrinology, Metabolism, and Diabetes, First Department of Pediatrics, National and Kapodistrian University of Athens Medical School, “Aghia Sophia” Children's Hospital, Athens, Greece; Division of Endocrinology and Metabolism, Center for Clinical, Experimental Surgery and Translational Research, Biomedical Research Foundation of the Academy of Athens, Athens, Greece; CeADAR: Ireland's Centre for Applied AI, University College Dublin, Dublin 4, Ireland; Lab of Computing, Medical Informatics, and Biomedical Imaging Technologies, Department of Medicine, Aristotle University of Thessaloniki, Thessaloniki, Greece; Division of Human Nutrition and Health, Wageningen University and Research, Wageningen, Netherlands; Lab of Computing, Medical Informatics, and Biomedical Imaging Technologies, Department of Medicine, Aristotle University of Thessaloniki, Thessaloniki, Greece; Lab of Computing, Medical Informatics, and Biomedical Imaging Technologies, Department of Medicine, Aristotle University of Thessaloniki, Thessaloniki, Greece; Department of Nutritional Sciences and Dietetics, School of Health Sciences, International Hellenic University, Thessaloniki, Greece; Department of Electrical and Computer Engineering, Aristotle University of Thessaloniki, Thessaloniki, Greece; Department of Electrical and Computer Engineering, Aristotle University of Thessaloniki, Thessaloniki, Greece; CeADAR: Ireland's Centre for Applied AI, University College Dublin, Dublin 4, Ireland; Division of Human Nutrition and Health, Wageningen University and Research, Wageningen, Netherlands; Department of Electrical and Computer Engineering, Aristotle University of Thessaloniki, Thessaloniki, Greece; Division of Human Nutrition and Health, Wageningen University and Research, Wageningen, Netherlands

**Keywords:** behavior, built environment, big data, childhood obesity, monitoring systems, physical activity, systems models

## Abstract

The relation among the various causal factors of obesity is not well understood, and there remains a lack of viable data to advance integrated, systems models of its etiology. The collection of big data has begun to allow the exploration of causal associations between behavior, built environment, and obesity-relevant health outcomes. Here, the traditional epidemiologic and emerging big data approaches used in obesity research are compared, describing the research questions, needs, and outcomes of 3 broad research domains: eating behavior, social food environments, and the built environment. Taking tangible steps at the intersection of these domains, the recent European Union project “BigO: Big data against childhood obesity” used a mobile health tool to link objective measurements of health, physical activity, and the built environment. BigO provided learning on the limitations of big data, such as privacy concerns, study sampling, and the balancing of epidemiologic domain expertise with the required technical expertise. Adopting big data approaches will facilitate the exploitation of data concerning obesity-relevant behaviors of a greater variety, which are also processed at speed, facilitated by mobile-based data collection and monitoring systems, citizen science, and artificial intelligence. These approaches will allow the field to expand from causal inference to more complex, systems-level predictive models, stimulating ambitious and effective policy interventions.

## Teaser

**Teaser Text**: Using large volumes and varieties of data—“big data”—can help to study the complex factors influencing obesity. This article explores the roles for big data in obesity research.

## Introduction

Obesity is one of the most important global health issues, owing to its association with significant morbidity and mortality, as well as increased public health costs. Overweight and obesity—as defined by abnormal or excessive fat accumulation that may impair health—has risen dramatically in prevalence during the last 4 decades in every global region (1–3). As of 2016, 13% of all adults had obesity and 39% had overweight; whereas, 28% of children had overweight or obesity as of 2017 (4). Overweight and obesity are risk factors for a broad set of noncommunicable diseases. Individuals with obesity were also recently estimated to be 1.5 times more likely to die of COVID-19 (5), because the associated immune system compromise, itself influenced by associated comorbidities, complicates treatment, recovery, and vaccination efficacy (6). Consequently, excess weight contributes to a substantial loss in disease-free years and avoidable deaths (3), which makes the identification of the major causes and drivers of obesity a primary concern. Although the “obesogenic environment” is a key predictor, specific causal factors related to the built and social environments remain underexplored. The global and uniform rise in obesity prevalence points to complex, multifactorial, and systemic causal factors (e.g., income, education, food quality), in contrast to the assumption that obesity is an individual's responsibility stemming from poor lifestyle choices (7, 8).

Although current approaches to explain obesity development consider many etiologic factors—from genetic to molecular to environmental levels—there is still an urgent need for tools and methods able to capture the complexity of the factors and systems contributing to obesity. Nearly 20 y ago, the Ecological Systems Theory was applied to obesity, leading to the ecological model of obesity, wherein an individual's weight status is heavily influenced by their social, economic, cultural, and environmental context (9). This model was further elaborated by the Foresight project, which identified 108 key variables relevant to energy balance, grouped into 7 themes: food production, food consumption, social psychology, individual psychology, physiology, individual physical activity, and the physical activity environment (10). These systems models, as well as others in obesity and nutrition (11–15), have offered tangible public health intervention points around which to shape policies and model scenarios aimed at obesity prevention and management, as well as a framework for modeling the concentric spheres of agents (e.g., individual, family, community, government, industry) that influence body weight gain (9, 16, 17). In addition, the *Lancet* Commission on Obesity has made critical leaps in systems thinking with landmark articles such as “The Global Syndemic” (11), delivering a transdisciplinary framework for addressing the interconnections between obesity, undernutrition, and climate change. Despite this, a holistic, data-driven understanding of *how* the complex milieu of the built and dietary environments and socioeconomic position (income, education, and health inequalities) affects individual behavior (nutrition and physical activity level) is still lacking. Although recent studies have made advances in capturing a wider range of systemic factors using mixed-methods approaches (18), static or traditional survey-based approaches fail to appropriately capture the complexity of factors and dynamic interactions between the individual and their environment (19, 20).

The adoption of *big data* creates the potential to generate synergies between biological and ecological approaches toward a better understanding of the etiology of obesity (21). Big data have the potential to identify intervention points for obesity prevention or management in vivo, across different environmental settings, as well as create data-driven causal maps of obesity determinants. A recent Delphi study sought to define big data in the context of obesity research, concluding that big data:

…is always digital, has a large sample size, and a large volume or variety or velocity of variables that require additional computing power. It can include quantitative, qualitative, observational, or interventional data from a wide range of sources (e.g., government, commercial, cohorts) that have been collected for research or other purposes, and may include one or several datasets. (22)

In this framework, volume concerns sample size, number of variables, or measurement occasions; variety concerns the types of data and numbers of variables; and velocity means that data are generated and processed in real-time or at speed—translating them into interfaces to make them readily usable (22). Moreover, the study noted that the variety component of big data in obesity research is more nuanced than its traditional definition within the big data domain, involving quantitative, qualitative, and observational data from a broad range of sources, e.g., clinical cohorts, census data, Geographic Information Systems (GIS) data, social media, advertising, and commercial sales data. To date, big data are under-utilized and under-exploited in obesity research. There are few widely available data sets concerning obesity that conform to the foregoing definition; few studies have used a wide variety of relevant variables, permitting multivariate investigations, and seemingly none have used velocity (22–24). Furthermore, it is not yet clear how obesity research can ethically use big data methods, although ethical frameworks are emerging from the literature (25, 26).

In this report, we investigate the potential for big data to support and expand current methodologies aimed toward systems approaches in the domain of obesity etiology. The complementarity and novel additions of big data to current obesity research approaches are discussed, as well as important risks and considerations when collecting such data, and the roles for domain and technological expertise.

## Traditional Compared with Emergent Approaches to Study Causal Relations in the Etiology of Obesity

The Ecological Systems Theory of obesity proposed 20 y ago added structure and form to our understanding of obesity as a chronic disease arising from biological, social, and environmental interactions (9). Combined with the arrival of the genomic and information ages, complex health (and biological) approaches to obesity etiology have emerged, including the Foresight model. In what follows, we discuss the contributions and pitfalls of current epidemiologic approaches to obesity research and highlight the ways in which big data may contribute.

### Epidemiologic: diet, socioeconomic, and food-environment drivers of obesity

Epidemiologic research in obesity has led to critical insights into obesity's noncommunicable disease sequelae and its disproportionate prevalence in socioeconomically disadvantaged groups in high-income countries. This line of research has also motivated hypothesis-driven research on the links between obesity and living environments; particularly as it relates to the drivers of food consumption, physical activity, and social psychology (27). Epidemiologic surveillance over time has also revealed the changing relation between these aforementioned factors and weight status over time; such as the associations between weight status and urban compared with rural living environments, or history of breastfeeding (28, 29). These insights have informed important public health interventions aimed at lowering inequalities in obesity, for example, programs targeting breastfeeding rates, school nutrition, or the reduction of sedentary behavior (30).

The large-scale WHO European Childhood Obesity Surveillance Initiative (COSI) has collected data from >300,000 children in Europe, leading to the development of a standardized monitoring system, and a variety of insights on, e.g., diet and physical activity patterns by country (31). The Healthy Behavior in School-Aged Children study, examining data from >130,000 adolescents from 29 European countries, was able to show that individual differences in physical activity could largely be explained by between-country differences in environmental variables, such as temperature, safety, household income, and public health policy (32). In the United States, the National Collaborative on Childhood Obesity Research has delivered critical research frameworks for childhood obesity, such as compendiums of physical activity codes relevant to youth, and measurement and surveillance needs for childhood obesity prevention from a variety of entry points (i.e., nutrition, physical activity, and food environments) (33–35). Despite these advances, these traditional data assessment methods lack the variety and granularity of data needed to explore a wider array of determinants—both group and individual—relevant to obesity. Classical epidemiologic approaches, such as dietary and exposure assessments followed by linkage to BMI, are strongly focused on the outcome of body size, rather than on complex associations between exposures, environments, and risk of obesity-related health complications.

In addition, BMI is often used as a metric for weight status; however, this is a proxy measure of adiposity and therefore an incomplete assessment of individual health status. Some individuals within the normal-weight range may display all characteristics of obesity, and those outside the normal range, not (36, 37). Importantly, the use of BMI and BMI cutoffs as a measure for weight status is problematic when comparing people with a different racial background, because body posture and build differ widely between these groups (36, 37). Moreover, these approaches also tend to orient (methodologically) from a biomedical lens. This means genes, nutrients, and foods, rather than social and environmental determinants (e.g., socioeconomic factors and the built environment), have taken the center stage (38, 39). Epidemiology also tends to side on inference of (health) outcomes based on behaviors and exposure, rather than data-driven prediction models of outcomes under differing or altered circumstances (40).

The more complex associations between exposures, environments, and outcomes are difficult to capture using traditional “offline” methods (food-frequency surveys, dietary intake recall) (41, 42). A systematic review of cohort studies that included both individual and ecological data purporting to examine the influence of food environment on obesity concluded that all available studies (*n* = 71) were of low quality and error-prone; and associations were predominantly null, failing to deliver meaningful conclusions or evidence (43). An additional scoping review on advanced methods linking dietary behaviors and the food environment uncovered only 5 studies with >100 participants that incorporated both Global Positioning System (GPS) and food intake data (44). None were transnational, and the period of data collection was a maximum of 7 d. The authors concluded that this paucity of data hinders the development of advanced modeling of food behaviors, such as agent-based modeling or complex-adaptive systems modeling. Incorporating environmental attributes in a manner that still enables the identification of causal relations, with attention to data veracity and reproducibility of variables, is a critical challenge for 21^st^-century epidemiologists. This is particularly the case for factors relevant to the social determinants of health as pertaining to obesity—for example, quality and accessibility of healthy foods or availability of athletics and recreation facilities.

### Big data: toward a more complex systems etiology of obesity

Big data are new and underexploited in obesity research. The most relevant contributions so far are from “found/real-world data” scraped for other purposes, such as social media, retail, and environmental sensing, rather than generated for specific obesity-related objectives or hypotheses (22). An agreed-upon scope and definition of big data for obesity research is both nuanced and still emerging (22). Moreover, an overview of case studies purposing to use big data in obesity research did not identify any studies using advanced sensors/apps for use by individuals or participants (45). The studies relied on post hoc analysis of 1-time administered surveys or electronic health records, in combination with data from external sources—e.g., land-value and census records (24). On the other hand, very recent advances have been made in using accelerometers to measure physical activity and its link to overweight and obesity risk, such as the International Children's Accelerometry Database Collaboration study (46). Gathering big data on obesity via mobile surveillance/monitoring has the promise to link relevant variables at the level of individuals and populations—allowing synergy between the biomedical and epidemiologic approaches. The emergence of new assessment methods means that individual or group-level data of large populations can be collected in real-time, allowing researchers to move away from average or self-reported values of food intake, mood, physical activity, or geolocation. This brings enormous potential to examine dietary behaviors more accurately over time and across contexts, which can lead to advanced predictive and causal models for planned interventions. The aforementioned Delphi study highlighted the need for the adoption of more advanced statistical, machine learning, artificial intelligence, and database methods to address complex hypotheses (22). These research avenues could include the influence of social media on food intake, or the prediction of weight status based on distinct environmental features—associations that are challenging to determine using traditional methods. Next, we outline 3 emerging areas where big data can be used in the obesity research context.

#### Mobile sensing

Mobile sensing and mobile health (mHealth) tools have the potential to continuously harvest behavioral and environmental data in real-time. This field is rapidly expanding (47), but remains underused in research contexts owing to the reliance on mainly commercially developed tools, and the lack of approved devices and systems for broad use in clinical trials. These approaches have the potential, although still challenging in a research context, to measure both physical activity and food intake (both self-reported and sensor-based), as well as collect data related to the built and food environments. These tools avoid the drawbacks of certain self-reported measures, and may also feed-forward either specific interventions or behavioral suggestions to the user, i.e., the use of “nudging” approaches to increase physical activity behavior (48). These strategies could be adopted not only using mobile phone–based sensors and apps but also via alternative means of data collection such as smartwatches, food-intake sensors, and other wearables (45, 49, 50). To date, a few pilot mHealth tools have been made available to gather information on food environments in a participatory way (51, 52). The European study DEDIPAC (DEterminants of DIet and Physical ACtivity) made steps toward capacity building for big data in the field, making progress, for example, in the adoption of physical activity sensor–derived data (53, 54). Measurement of physical activity over time among diverse groups is therefore feasible; however, broader efforts to incorporate these measures in real-time, in combination with social/environmental determinants of physical activity, have not yet been achieved.

#### Citizen science

The approach of citizen science uses large and engaged populations as data providers, involving the participant in the whole scientific process. This approach not only facilitates “feeding-forward” of health information to participants, but also exploits and/or enhances their knowledge base of science and health (55). Citizen science tools for health monitoring in the public domain are generally developed in controlled populations, followed by dissemination to the public; or involve crowd-sourced data contributions or scraping of existing databases. In the health care domain, projects include SCAMPI (the Smart City Active Mobile Phone Intervention), a mobile intervention tool to promote physical activity among sedentary adults (56), and Big data against childhood Obesity (BigO), discussed in detail in what follows. The Our Voice project, a mobile community engagement tool, was deployed in 5 countries and collects photos and voice memos from individuals on the theme of physical activity and food access (57). A major drawback to a citizen science approach from a research perspective, particularly as linked to health-related behaviors, is that it is accompanied by an uncontrolled sample population. Therefore, uncontrolled data collection protocols and reliance on self-reported measures of health make it challenging to derive valid associations (58). The use of traditional statistical approaches on this type of data can be erroneous. New statistical approaches need to be developed and used that consider selectivity biases, the sparseness of data, and noise. These aforementioned issues have been recognized by Eurostat and all European Union national statistical authorities, but can take inspiration from success in other domains, for example in environmental monitoring of biodiversity (59, 60).

Moreover, the use of citizen science tools may be biased to urban, relatively wealthy, highly educated individuals, although this bias is also a known pitfall in food intake and nutrition survey responses. The steady increase in smartphone use in all global regions, particularly among younger users, could overcome this with appropriate dissemination efforts (61).

#### Artificial intelligence

The rapid evolution in the technological development of artificial intelligence, including machine and deep learning, is moving research beyond data collection and toward knowledge extraction and prediction. Artificial intelligence promises to mine purchase, Internet, and social media data in an aggregated manner, allowing analysis, visualization, and prediction from individual and group-level big data. In the context of obesity, this technique has the promising potential outcome of deep learning–derived prediction of population behavior and weight status, based on environments or proposed interventions. Artificial intelligence has the potential to overcome some of the aforementioned sampling biases in citizen science–based approaches, eventually leading to the automatic fitting of prediction models and the identification of modifiers and confounders—allowing the prediction of the outcomes of health interventions (62, 63). So far, this type of prediction has been largely the domain of tech giants such as Facebook, IBM Watson, Google, and Amazon. For example, the power of Google's data access and intelligent computing capacity is being combined in “Google Health,” which aims at predicting and treating a variety of health issues. Critics are concerned about this form of industry research, including a lack of standards for ethical accountability and informed consent (63). In the public domain, the predominant use of artificial intelligence as it pertains to diet and eating behavior has been in computational neuroscience, with emergent applications aimed at automating eating behavior analyses (64–67). The development of complex adaptive systems and agent-based models of complex drivers and determinants of eating behavior within varied food environments have the potential to generate rich and accurate predictions of behavior (68–70). However, the volume and variety of data required to build reliable and accurate models so far remain largely unavailable in publicly available data sets owing to data-sharing challenges (44). The next step for computational researchers concerned with dietary behavior will be to apply artificial intelligence techniques to big (rich) data comprising food accessibility, preference and choice (i.e., dietary patterns), eating behavior (i.e., eating rate, satiety, satisfaction and fulfillment, setting), health profile, psychology, socioeconomic status, and various aspects of the built environment—leading to insights into obesity etiology and guiding targeted prevention measures. As until very recently these techniques and data have largely remained the property of industry, there must be increased attention paid to responsible knowledge transfer, in part via public–private partnerships, and the required ethical frameworks (71).

## How Can Big Data Complement Traditional Research Approaches?

The advanced means of data acquisition described already are only beginning to be used in research and public health contexts. Although most researchers are no doubt aware of their promise, epidemiologists concerned with obesity and public health are at risk of lagging behind big tech or profit-driven data acquisition pursuits. The European Centre for Disease Prevention and Control has promoted a policy on the use of big data in epidemiology (72, 73). This approach will not only allow the pursuit of cross-cutting hypotheses but improve data quality and make resource-intensive surveillance efforts more efficient (74). Here, we discuss 3 thematic areas where the big data tools already described can complement traditional approaches in obesity research: *1*) food intake and eating behavior, *2*) social food environments, and *3*) the built environment and physical activity. All 3 themes have the potential to utilize mobile sensing, citizen science, and artificial intelligence approaches described in the previous section, complementing and aiding traditional methods. Representative examples of specific research questions and a subset of data needs per theme are proposed in **Table 1**. The wide variety of big data types are not captured here exhaustively; this is intended to illustrate potential research avenues and stimulate already-existing disciplinary framing to adopt aspects of big data. Many variables sit across themes and their exploration will only serve to further benefit systems thinking on obesity.

**TABLE 1:** Examples of big data contributions to obesity research, by research dimension^1^

Research dimension	Research question	Data needed	Potential data sources	Methods needed	Potential outcomes
Food/nutrient intake and eating behavior—nutritional theme	How do food characteristics (taste, texture) interact with social setting to control satiety?	Rich food composition and features database [volume/variety] Objective measures of social setting: eating regularity and duration, family, school, and peer eating activity [volume/variety] Timing of food intake and objective/subjective satiety measures in real-time, not, e.g., daily average [velocity]	National food consumption surveys EFSA food composition database (97) Publicly available nutrition and food composition applications—as documented (98, 99)	Automated or semiautomated food profiling/food composition spectrometry in FAIR databases Automated meal sensing (wearable sensors/AI-driven meal picture analysis, observational restaurants) Mobile-based surveys	*Research:* integrated biological, economic, and social sciences understanding of the determinants of food intake *Innovations:* novel food types with high satiety index *Policies:* health policy promoting specific eating practices in schools, families, and workplaces, according to healthy and sustainable FBDGs; tailored to context
Social food environments—psycho-social theme	What role does food and diet portrayal in digital media (advertising and social media) play in food and beverage intake?	Real-time food-intake assessments and subjective/objective determinants of satiety [volume, velocity] Social media and real-world advertising exposure logs [variety] Source of digital media advertising/marketing (e.g., via industry or from users themselves)	National food consumption surveys Social media content, behavior and network data via APIs/web scraping Regional/local census and statistics bureaus	Mobile/sensor-based food intake measurements Citizen/crowd-sourced mapping tools Monitoring of digital advertisements (e.g., via Web browser/application plugins)	*Research:* role of social media, influence, and social networks in obesogenic behaviors *Innovations:* effective healthy diet–targeted advertisement campaigns/nudging *Policies:* food advertisement policies for more targeted restriction/promotion of certain foods
Built environment—environmental theme	What combinations of features of the built environment influence physical activity levels and weight status?	GIS-derived points of interest per region [volume/variety] Socioeconomic, education, ability, and health characteristics [volume/variety] Real-time, GPS-correlated physical activity measurement [volume/velocity]	National food consumption surveys GIS/Google/Foursquare USDA Food Environment Atlas Regional/local census and statistics bureaus (e.g., Eurostat) Map the Meal Gap (US) (100) Food Environment Atlas (US) (101) Global Physical Activity Observatory (102)	Computational tools for scraping environment characteristics, linked in real-time to user activity Mobile-based tracking of anthropometry and activity levels	*Research:* relation between built environment, physical activity, health-related behaviors, and obesity prevalence *Innovations:* interactive intervention/policy design tools; tools for real-time monitoring of policies and interventions *Policies:* toward healthier, physical-activity-promoting, and accessible urban and suburban environments

1 AI, artificial intelligence; API, Application Programming Interface; EFSA, European Food Safety Authority; FAIR, findable, accessible, interoperable, reusable; FBDG, food-based dietary guideline; GIS, Geographic Information Systems; GPS, Global Positioning System.

### 1. Food intake and eating behavior—the nutritional theme

Implementing effective nutrition interventions for obesity prevention requires a more complete understanding of individual (biological) and food (nutritional, chemical, and physical) factors that influence eating behavior and appetite (75–77). These factors include both food characteristics (taste, texture, food matrix, energy density, food form, satiation and satiety, satisfaction, and fulfillment) and individual physiology (metabolic and endocrine responses, body composition, and level of physical activity). Big data have the potential to complement single-study-derived insights into food intake, eating rate, and their link to satiety. These can be linked to relevant bio-social individual characteristics such as history of breastfeeding, health comorbidities, and subjectively reported satiety and motivations for food choice. For example, mobile-supported objective measurement of eating rate was recently found to be associated with higher BMI among adolescents (66, 67, 78). Libraries of food characteristics can be linked to genomics and metabolomics data sets, and/or advanced food-intake sensors, as well as data relevant to satiety. The timing of food consumption and the time occupied with food ingestion could also be linked to the level of intake and physical activity. Such integrated big data approaches would greatly enhance our understanding of how food intake is influenced or can be modified and can also help to overcome some of the issues inherent in relying on the metric of BMI as a measure of weight status, moving toward a more holistic assessment of health risk on both an individual and a population level.

### 2. Social food environments—the psychological theme

Branches of nutrition science and consumer behavior are typically concerned with the social determinants of food intake and eating behavior, including the social environment (e.g., eating alone compared with eating in a group) (79), in addition to availability, accessibility, affordability, and exposure to food advertising (80). Big data allow investigation and modeling of both the real-world and the social-media/digital food environments as determinants of food intake, taking into account cultural, racial, and socioeconomic background and the surrounding food environment, building on food intake data described in theme 1. Research must address the social drivers and motives that influence individuals in the mixed “real” and digital food environment, and how these drivers influence food preference, intake, satiety, and dietary profiles. Monitoring social dietary behaviors via methods more advanced than traditional surveys or observational studies will become possible, such as observational purchase data from supermarkets and restaurants or surveillance of food- and diet-related individual interactions with social media. This dimension also concerns the influence of national or regional agricultural policy and subsidies on food availability and affordability and the generation of data infrastructures able to categorize and compare these factors in relation to epidemiologic outcomes. The INFORMAS healthy food environment policy index is a critical step toward a framework within which big data on food environments can be monitored (81).

### 3. Built environment—the environmental theme

The design and implementation of effective public health policy addressing obesity require an understanding of what features, or combinations of features, in the built environment influence food availability and obesogenic behaviors—both food intake, as outlined in the first 2 themes, as well as physical activity. Studies of these associations typically use limited cohorts or data of insufficient granularity and variety (e.g., census data), although recent studies have made progress in linking neighborhood composition to body composition and movement behaviors (82). Big data will allow for data-driven testing of the ecological model of obesity, linking objective aspects of behavior (step and accelerometry data, GPS coordinates of locations visited) to weight status, demographic data, and detailed characteristics of the built environment such as densities of food-related points of interest and rich data on their offerings and affordability (via geo-sensing and digital mapping).

### Summary

Table 1 outlines potential research questions in each of these dimensions, the required data, the big data and traditional complementary methods needed, and potential outcomes in the realm of innovation or public health policy. These questions may serve as a conceptual starting point for epidemiologists, health professionals, and social scientists seeking to use big data in obesity research.

## *How* Can Big Data Contribute to Evidence-Informed Policy Making? Case Study of the BigO Project

Given that many forms of data known to be highly relevant to obesity are currently out of reach of traditional epidemiologic research (e.g., mobile/GPS data; social media and digital advertisement data concerning the social food environment; rich mapping of food environments), immediate steps must involve improved interdisciplinary collaboration of computer and data scientists and engineers, led by nutrition and medical domain-experts. Big data in obesity have the potential to examine intervention outcomes or observational studies and expand causal inference of ecological associations to more complex, systems-level multivariate models, disentangling individual-level and environmental determinants in specific groups and contexts. Big data collection tools have the potential to test associations using an unbiased representation of multiple variables of interest, such as aspects of the built environment and physical activity. More excitingly, the use of big data also expands the possibility for much-needed predictive models, predicting, for example, how changes to the built environment could change physical activity and diet-related behaviors, or how changes in behavioral risk factors for obesity affect prevalence.

**FIGURE 1:**
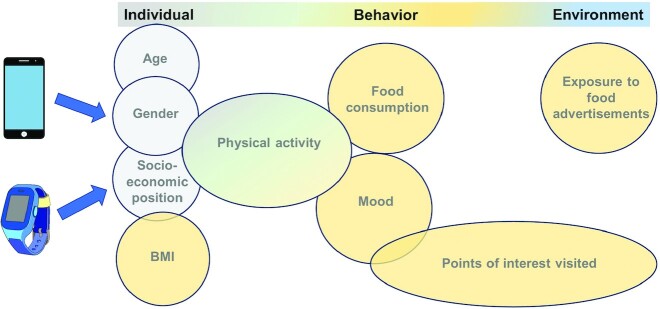
Variables in a mobile citizen-science approach for obesity. An example data map, modeled after the BigO system, of the types of variables pertinent to obesity able to be collected via mobile health approaches. These variables pertain to individual characteristics (demographic, anthropometric), individual behavior (physical activity, food consumption, mood), and the built environment (points of interest visited, food advertisement exposure).

Responding to a lack of effective data-driven policies for the prevention of childhood obesity, BigO: Big data against childhood obesity, a Horizon2020 project, was launched in 2016. The project took action to address the lack of behavioral data processed in real-time (velocity), as well as the need for comprehensive tools for stakeholders seeking to design and implement policies. Using an mHealth and citizen science approach, BigO built a technology platform to capture environmental features and link them to individual behavior and weight status of children. The project is intended as a starting point for assessing the link between weight status and the built environment using data gathered from the platform, as well as a blueprint for future projects aiming to gather multivariate big data on diverse behaviors related to individual and environmental characteristics. To our knowledge, BigO is the only program to have collected data on physical activity and the built environment as related to weight status (BMI *z* score) within the same individual, as well as subjective/qualitative measures of health and well-being, in multiple countries and socioeconomic groups and across all BMI strata (83, 84). **Figure 1** shows the scope of the data collected about individual factors, behaviors, and environmental characteristics.

The project involved >5500 children who acted as “citizen scientists,” submitting data via a smartwatch or smartphone application. The children were spread across 4 European countries, recruited via schools and obesity clinics, with weight status being both self-reported and clinic/school-measured reports. The BigO program developed a user-friendly mHealth app (MyBigO), because it was intended for use by children from 9 y of age onwards. To achieve this, a rigorous testing procedure was adopted consisting of 6 levels: code quality testing, unit testing, black box testing, integration testing, load testing, and beta testing (assimilation of both system and acceptance testing). This testing procedure proved effective because the app was widely accepted by both children and obesity clinicians, and successful at collecting the intended breadth and volume of data (85–87). The collected data are big in *variety*: GPS data, accelerometry, mood questionnaires, meal photos, and food advertisements encountered in their offline environment are recorded alongside anthropometry. They amount to big *volume*: the >5500 children submitted >100,000 meal photos to the platform between 2018 and 2020, >31,000 mood questionnaire responses, and >130,000 d of GPS and accelerometry recordings. The data were also processed at *velocity*: data were processed near real-time into dashboards to monitor, for example, the average activity counts per neighborhood of different municipalities, in relation to features of the built environment such as food outlets and physical activity locations. Publicly available e-dashboards were constructed, which can be found at http://bigo.med.auth.gr:3838/. Password-protected dashboards were available for clinic and school use, and are intended as health monitoring tools for the medical staff within schools and clinics. The public health dashboard is publicly available and is intended for eventual public health use as a policy planning tool. This technical platform fills a major gap in data-driven policy planning for obesity prevention.

Rather than starting from biological causal hypotheses of physical activity and weight status, data in BigO were gathered among a large and diverse population, which will be followed by hypothesis-generating models linking environmental characteristics, physical activity, and weight status. Making objective measurements of physical activity, linked to aspects of the built environment and weight status, all within the same individual, and taking into account the granular temporality of food intake and physical activity, promises to reveal dynamic behavior of individuals and populations in real-time (83, 88, 89). The system also has the potential to function as an intervention tool, feeding-forward rewards to incentivize the use of the app, which may have the added effect of incentivizing healthier behaviors. In this line, BigO was also conceived and implemented for use in obesity clinics to track, manage, and eventually explain varying dimensions of weight loss on an individual level (85). Further exploitation of these data in the clinical context will provide insights into individual factors relevant to weight loss rate, maintenance, and recidivism in response to specific interventions.

Research projects and initiatives such as BigO build on the interplay between individual, social, and environmental determinants of behavior to help guide the development, monitoring, and evaluation of preventative strategies. To make such initiatives maximally relevant to public health policy, there is a need to exploit the data generated to feed advanced predictive models of individual and population determinants of BMI and eating patterns. The end goal for these types of initiatives is the development of contextually effective, ambitious data-driven policies targeting a large segment of the obesity map: individual food intake and physical activity, the school environment, and the built environment.

## Limitations and Considerations

Despite its promises, several important caveats and limitations surround the use of big data, particularly when tracking multiple individual characteristics or behaviors across time and space. These limitations are discussed and supplemented with experience from the BigO project and presented further in **Table 2**.

**TABLE 2:** Overcoming challenges to the use of big data in obesity research^1^

Challenge	Potential solutions
Privacy	Automatic local/offline prechecks built into apps for identity-violating content Process location locally to geohashes or other aggregated values Assign tiered data access to investigators Involve domain experts/researchers, educational and citizens’ groups (end users) in privacy co-design
User bias	Align with city-lab and health equity and health promotion initiatives Specific targeting of communities of lower socioeconomic position via schools, clinics, and community organizations Control for access to health care
Veracity	Domain-specific standards for specific types of noisy, error-prone data and volume reduction Open-access, user-friendly tools to facilitate implementation of data standards
Legacy and FAIR data	Integrate big data in nutrition and public health with emerging research infrastructures Align emerging data and projects to the European Open Science Cloud and surveillance efforts [e.g., INFORMAS (81, 103)]
Industry vs. research interest	Ethical forums, conferences, and guidelines for big data industry–academic partnerships Research training in big data techniques and communication with technical experts

1 FAIR, findable, accessible, interoperable, reusable.

### Privacy concerns are significant when using mobile tools, especially among children

Although the General Data Protection Regulation, which has brought about a guarantee of location privacy, provides procedures for obtaining informed consent for the use of GPS in the age of smartphones, this is an emerging area still subject to varying degrees of stringency depending on country and institution (90). The potential involvement of young, vulnerable data contributors (i.e., children and adolescents), as well as centralized, cross-border data collection schemes, further complicates matters. A powerful and innovative approach was developed within BigO to support privacy based on spatial aggregation of behavioral data, described in Diou et al. (83). Issues related to the location were resolved by processing location data locally in each mobile device before sending them to the central server and grouping them into larger geographic zones, or “geohashes.” However, all data collected by children, such as pictures related to their food intake, were at risk of violating individual privacy. For instance, some children sent photos of themselves to the server. These data were preprocessed manually, and all images deemed irrelevant or identity-revealing were deleted from the system. However, this procedure was time-consuming, and it is not a scalable solution; future efforts to incorporate automatic image recognition should help to overcome this challenge.

### Big data warehouse architecture should implement privacy-aware protocols for monitoring and storing personal data related to the behaviors of a potentially vulnerable population

Individual health status and behavioral data sets must be protected and kept private. On the other hand, these data must remain analyzable and ideally reusable to extract useful knowledge that advances research and practices. The aim is to create an environment where private and sensitive data can be analyzed without revealing the identity of individuals. In BigO, an n-tier architecture to deal with the data analysis was proposed, with various defined investigator roles, where each role had a very limited view of the data, together with anonymity procedures. There also exists the challenge of acting on anonymized data collected from vulnerable populations, where, e.g., child protection or welfare is a concern. The development of data analysis pipelines for either encrypted or anonymized data remains a complex task.

### In terms of sampling, without attention to recruitment and dissemination, citizen science tools risk including only the already healthy and wealthy, groups which are the least affected by overweight and obesity

Managing bias in sample populations in big data approaches which incorporate large populations is not something that has yet been systematically addressed in the big data approaches toward obesity to date. This issue exacerbates the already present challenge of determining causality from uncontrolled data collection. Sampling bias was a particular concern for data gathered in the BigO project, where many of the participating schools were fee-paying and richly resourced, whereas the clinical population represented children from a much wider range of socioeconomic backgrounds, with varying access to health care depending on the country.

### Concerning the scope of data extracted, it is not always the case that more volume means more quality

Filtering out the appropriate level of data from big data collection methods (e.g., physical activity data sampled by the second or minute) is critical to reaching reliable conclusions. Approachable or standardized methods for both data reduction and managing data veracity are needed (e.g., low GPS accuracy, missing data from an individual not carrying their device, or errors in translation from signal to behavior). When considering large populations, engaging individuals over long periods of time to contribute data poses many logistical and tactical challenges, as does coping with the ensuing mismatched or missing data. In the BigO project, domain expertise, gathered via a Delphi study, was essential to prioritize the most relevant variables to childhood obesity, and considerable effort was made to maximize user engagement (91). Despite the high volume of data that can be captured, many critical factors relevant to obesity remain unexplored. This is evidenced by the many remaining unmapped systems-level domains and nodes (as mapped by the Foresight report) (92) relevant to obesity, and they are not yet incorporated into big data frameworks. For example, physiologic factors (satiety, extent of digestion and metabolism, predisposition to reduced physical activity level), societal influence (peer pressure, parental control), and individual physical activity (degree of physical activity education, parental modeling of activity, physical barriers to movement) (92).

### Ensuring a legacy for big data collected in public-funded research projects is also an emerging concern

Considerable amounts of quantitative, qualitative, and observational or intervention data already exist, collected for research or other purposes. Ensuring that these data are findable, accessible, interoperable, reusable (FAIR) and using them to feed existing models should therefore be a priority. Of particular concern is ensuring the interoperability and reuse (FAIR-ification) of data while maintaining individual privacy rights. The legacy of data, particularly when gathered in time-limited research projects, can also be threatened. Alignment with existing and emerging health and digital science research infrastructures, as well as conforming to the European Open Science Cloud is a first step to overcoming some of these legacy concerns. Alignment and FAIR-ification can also ensure the reuse of big data collected in the framework of health and obesity research for other domains to which it is of tremendous value, e.g., urban design, consumer behavior, and educational sciences. Ensuring the interoperability of big data in obesity is of particular interest given the emerging linkages between obesity and COVID-19. Big data mobile surveillance tools could pivot to assess the impact of lockdowns on diet, physical activity, and well-being, or ongoing efforts linking the built environment and obesity can be aligned to infection mapping (93).

### Balancing obesity domain expertise with the required technical and computational expertise—academic or not—remains a key challenge in academic and policy pursuits using big data

The data analytics industry holds a monopoly on tool development, which risks overtaking nutrition and obesity research. If academic nutrition and epidemiology experts do not undertake action to shape big data use in obesity research through the lens of their domain expertise, critical health policy insights may risk being biased by profit-driven motives (25, 26). Moreover, domain experts in nutrition and epidemiology require training in the incorporation of big data, and hypothesis-generating rather than hypothesis-testing approaches. The development of agri-food policy over the past half-century has been fostered by economically driven industry motives and has (unintentionally) contributed to the overweight and obesity epidemic and its associated health costs (94–96). Now there is, therefore, a critical opportunity to change course to ensure the health of present and future generations, leveraging scientific and policy interest in the value of public goods to increase public health, well-being, and equity.

## Conclusion

The collection of big and rich data on diet-related behaviors and physical activity, concerning the built environment, is critical and timely to arrive at a more advanced and integrated understanding of the inherently complex etiology of obesity. Adoption of big data approaches has not been rapid in the field of obesity, as there remains ambiguity regarding its integration into traditional approaches. The Foresight model has provided a critical first framework to conceptualize and organize the multitude of factors determining energy balance; the data-driven collection and exploration of these have the potential to lead to new hypotheses for traditional biomedical approaches (e.g., more advanced understanding of food properties’ effects on metabolism) or epidemiologic approaches (e.g., multivariate, longitudinal models of environmental determinants of physical activity levels and food intake). The multicausal nature of the obesity epidemic makes it challenging to disentangle and quantify the influence of biology, behavior, and built environment, calling for an approach wider in scope than traditional epidemiologic methods alone. This can lead to advanced predictive models of the effects of public health interventions, and the ability to prioritize the most effective interventions for a given context. Critical attention and consideration must be paid to data privacy, management, reusability of data, and balancing industry with research interests to facilitate the use of big data as a public research resource. Investing in and adopting tools to allow the mapping of behavior about the social and built environment will provide a much-needed evidence base for the design and implementation of effective and contextually tailored public health policies and strategies aimed at the prevention of overweight and obesity.

## Notes

Supported by Horizon2020 Framework Programme project grant agreement no. 727688: “BigO: Big data against childhood obesity.”

Author disclosures: the authors report no conflicts of interest.

Abbreviations used: FAIR, findable, accessible, interoperable, reusable; GPS, Global Positioning System; mHealth, mobile health.
